# Advanced Mass Spectrometric Methods for the Rapid and Quantitative Characterization of Proteomes

**DOI:** 10.1002/cfg.159

**Published:** 2002-04

**Authors:** Richard D. Smith

**Affiliations:** Environmental and Molecular Sciences Laboratory MSIN: K8-98 Pacific Northwest National Laboratory P.O. Box 999 Richland WA 99352 USA

## Abstract

Progress is reviewed towards the development of a global strategy that aims to extend the sensitivity, dynamic range, comprehensiveness and throughput of proteomic measurements based upon the use of high performance separations and mass spectrometry. The approach uses high accuracy mass measurements from Fourier transform ion cyclotron resonance mass spectrometry (FTICR) to validate peptide ‘accurate mass tags’ (AMTs) produced by global protein enzymatic digestions for a specific organism, tissue or cell type from ‘potential mass tags’ tentatively identified using conventional tandem mass spectrometry (MS/MS). This provides the basis for subsequent measurements without the need for MS/ MS. High resolution capillary liquid chromatography separations combined with high sensitivity, and high resolution accurate FTICR measurements are shown to be capable of characterizing peptide mixtures of more than 10^5^ components. The strategy has been initially demonstrated using the microorganisms *Saccharomyces cerevisiae* and *Deinococcus radiodurans.* Advantages of the approach include the high confidence of protein identification, its broad proteome coverage, high sensitivity, and the capability for stableisotope labeling methods for precise relative protein abundance measurements.

*Abbreviations*: LC, liquid chromatography; FTICR, Fourier transform ion cyclotron resonance; AMT, accurate mass tag; PMT, potential mass tag; MMA, mass measurement accuracy; MS, mass spectrometry; MS/MS, tandem mass spectrometry; ppm, parts per million.


**Comparative and Functional Genomics**
*Comp Funct Genom* 2002; **3**: 143–150.
Published online 11 March 2002 in Wiley InterScience (www.interscience.wiley.com). **DOI:** 10.1002/cfg.159

**Conference Review**

### Advanced mass spectrometric methods for the rapid and quantitative characterization of proteomes

Richard D. Smith*
*Environmental Molecular Sciences Laboratory, Pacific Northwest National Laboratory, PO Box 999, Richland, WA 99352, USA*

*Correspondence to: Environmental and Molecular Sciences Laboratory, MSIN: K8-98, Pacific Northwest National Laboratory, P.O. Box 999, Richland, WA 99352, USA.*

### Abstract

**Progress is reviewed towards the development of a global strategy that aims to extend the sensitivity, dynamic range, comprehensiveness and throughput of proteomic measurements based upon the use of high performance separations and mass spectrometry. The approach uses high accuracy mass measurements from Fourier transform ion cyclotron resonance mass spectrometry (FTICR) to validate peptide 'accurate mass tags' (AMTs) produced by global protein enzymatic digestions for a specific organism, tissue or cell type from 'potential mass tags' tentatively identified using conventional tandem mass spectrometry (MS/MS). This provides the basis for subsequent measurements without the need for MS/MS. High resolution capillary liquid chromatography separations combined with high sensitivity, and high resolution accurate FTICR measurements are shown to be capable of characterizing peptide mixtures of more than $10^5$ components. The strategy has been initially demonstrated using the microorganisms *Saccharomyces cerevisiae* and *Deinococcus radiodurans*. Advantages of the approach include the high confidence of protein identification, its broad proteome coverage, high sensitivity, and the capability for stable-isotope labeling methods for precise relative protein abundance measurements.**

***Abbreviations*: LC, liquid chromatography; FTICR, Fourier transform ion cyclotron resonance; AMT, accurate mass tag; PMT, potential mass tag; MMA, mass measurement accuracy; MS, mass spectrometry; MS/MS, tandem mass spectrometry; ppm, parts per million.** **Copyright © 2002 John Wiley & Sons, Ltd.**

**Keywords:** **Fourier transform ion cyclotron resonance; mass spectrometry; proteome; proteins; *D. radiodurans***

### Introduction

The ability to study how the components of a biological cell or organism change and interact following a perturbation provides a foundation to understand the function(s) of its component parts, and ultimately how the system operates. Reaching this goal will require instrumental and computational methods that identify systems-level responses that recognize genes or gene products that are sensitive to changes in the environment of the cell or organism. Thus, considerable attention is now focused on the proteome, the complement of proteins expressed by a particular cell, organism or tissue at a given time or under a specific set of environmental conditions.

The currently existing proteome analysis capability is predominantly based upon protein separations using two-dimensional polyacrylamide gel electrophoresis (2D PAGE). While 2D PAGE is capable of resolving thousands of proteins, proteome coverage is problematic for proteins that have very high or low isoelectric points ($< \sim 3.5$ and $> \sim 9.5$), extremes of molecular weight, and membrane proteins, which typically account for more than half of all the proteins expressed within a cell. It has been shown that the number of spots is often poorly correlated with the number of different proteins detected, since a single gene can give rise to multiple spots [4] due to co- and post-translational modifications, degradation intermediates and alternative expression (e.g. alternative splicing of mRNAs,

Copyright © 2002 John Wiley & Sons, Ltd.

translational frame shifts). The sensitivity of 2D PAGE is generally limited to femtomole levels [11,14] by the need to visualize the protein spot on the gel and its subsequent processing and analysis primarily using mass spectrometry (MS) [10,14,15]. Finally, the precision of protein abundance determinations using 2D PAGE is based on comparison of protein spot intensities, limiting the capability for discerning subtle differences in protein abundances for large numbers of proteome-wide measurements [12].

Here we review the technological basis and progress towards a global proteomics strategy that aims to provide large improvements in sensitivity, dynamic range, comprehensiveness and throughput based upon the use of peptide 'accurate mass tags' (AMTs) [3]. The two-stage strategy exploits high resolution capillary LC separation combined with Fourier transform ion cyclotron resonance mass spectrometry mass spectrometry (FTICR) to validate peptide AMTs for a specific organism, tissue or cell type [2,6,9]. AMTs are peptide biomarkers that are used to confidently identify a unique protein based on the high mass measurement accuracy provided by FTICR. The identification of these biomarkers using tandem mass spectrometry (i.e. MS/MS) provides the basis for second-stage high throughput studies using only AMTs to identify and quantify the proteins expressed within a cell system. Key attractions of the approach include the feasibility of completely automated high confidence protein identifications, extensive proteome coverage, and the capability for exploiting stable-isotope labeling methods for high precision abundance measurements [13]. Additional developments, including the use of multiplexed-MS/MS capabilities [6], methods for dynamic range expansion of proteome measurements, [1] and multi-stage separations also promise to enable more focused analyses and further extend the quality of measurements and their extension to more complex proteomes.

### The new proteome measurement technology and applications

The aim of our strategy for proteome analysis developed over the last several years is to exploit a combination of instrumental and methodological approaches to provide broad proteome coverage, high sensitivity and the capability for greatly increased throughput compared with conventional technologies [13]. After initial cell lysis the recovered proteins are enzymatically digested into peptide fragments (e.g. using trypsin) to produce tens to hundreds of potentially detectable peptides (and modified peptides) from each protein, and perhaps $10^5$ to $>10^6$ in total (depending upon proteome complexity, the dynamic range of the measurements etc.). This complex peptide mixture is then analyzed by combined high resolution capillary separations-FTICR.

The extent of proteome coverage for any approach depends substantially on the achievable dynamic range of the MS measurements, which in turn depends significantly upon the resolution (or peak capacity) of the separation step(s) preceding MS analyses and any overall constraints due to sensitivity. As shown by the example in Figure 1, the dynamic range obtainable in a single FTICR mass spectrum exceeds $10^3$. The most highly abundant peptide eluted over a series of spectra, while low abundance peptides are often detected within a single spectrum. Therefore, the effective dynamic range for detection of peptides is $\sim 10^4$. Furthermore, if one's aim is protein identification, then a significant (perhaps 10-fold) increase in effective dynamic range will result due to the variable ESI or detection efficiency for different peptide sequences, and we estimate that the dynamic range achieved here initially is approximately $10^4$ to $10^5$ [9].

The power of MS for protein identification derives from the specificity of mass measurements for either the intact peptides or their fragments after dissociation in MS/MS measurements, and is implicitly based upon the relatively small number of possible peptide sequences for a specific organism compared to the total number of possible sequences (see Table 1). The distinctiveness of peptide sequences increases with size, but in practice the utility of increased size for identification is mitigated by the increased likelihood that a peptide will be unpredictably modified. Though much smaller than the number of possible sequences, the number of potentially distinguishable peptide *masses*, given sufficient resolution and accuracy, also dwarfs the number of predicted peptides from any organism. As shown in Table 2, an ideal tryptic digestion of all yeast proteins would produce 194 239 peptides having masses between 500 and 4000 Da, the range typically studied by MS. Of these, 34% are unique at $\pm 0.5$ ppm MMA. (A larger fraction is unique if constrained by additional information resulting

Copyright © 2002 John Wiley & Sons, Ltd. 

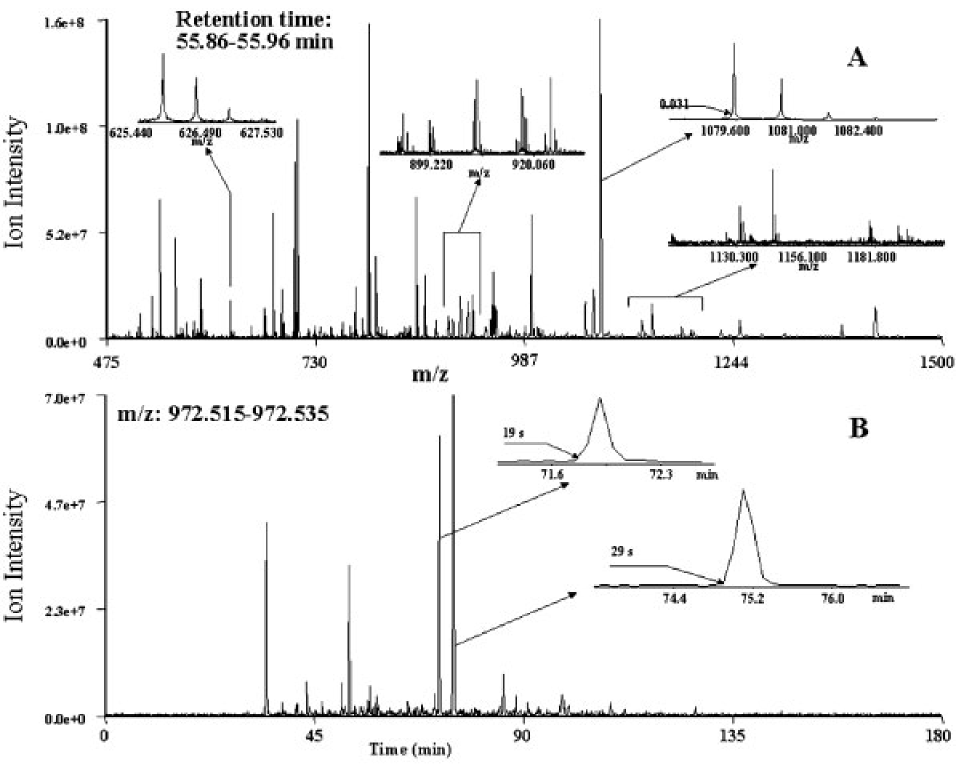

**Figure 1.** Illustration of the high quality of FTICR data (top) and the LC summed ion signal intensity during the separation (bottom) for a capillary LC-FTICR analysis of a soluble yeast protein digest. The separation was conducted at 10 000 psi to afford a LC peak capacity of ~800, as illustrated by the narrow peak widths (bottom). The high magnetic field FTICR instrumentation simultaneously provides high dynamic range, resolution, sensitivity and mass measurement accuracy. As a result, more than 100 000 species can be distinguished in a single analysis from any prior sample fractionation steps or the use of LC elution times.) These distinctive peptide masses would cover 98% and 96.6% of all predicted *S. cerevisiae* and *C. elegans* proteins, respectively.

Thus, given sufficient MMA, a peptide mass measurement can often be confidently attributed to a single protein within the constraints provided by a single genome sequence and its predicted proteome (i.e. serve as an accurate mass tag; an AMT). The AMT strategy obviates the routine need for MS/MS for peptide identification, and thus reduces sample requirements. Since the masses of many peptides

Table 1. Number of possible peptides and number predicted for three organisms from digestion with trypsin

| | **Possible** | | **Predicted number of peptides**[3] | | |
|---|---|---|---|---|---|
| **Length** | **Sequences**[1] | **Masses**[2] | ***D. radiodurans*** | ***S. cerevisiae*** | ***C. elegans*** |
| 10-mers | $10^{13}$ | $2 \times 10^{7}$ | 3471 | 11 275 | 30 623 |
| 20-mers | $10^{26}$ | $7 \times 10^{10}$ | 1292 | 3463 | 9475 |
| 30-mers | $10^{39}$ | $2 \times 10^{13}$ | 494 | 1278 | 3602 |
| 40-mers | $10^{52}$ | $1 \times 10^{15}$ | 195 | 405 | 1295 |

[1]Assumes 20 possible distinguishable amino acid residues.
[2]The number of peptides of length **r** potentially distinguishable by mass based upon the number of possible combinations of n different amino acids. $(n+r-1)!/r!\ (n-1)!$. The actual number of possible masses is somewhat smaller due to some mass degeneracy. The number of distinguishable peptides in actual measurements depends upon the MS resolution.
[3]Predicted from the identified open reading frames and applying the cleavage specificity of trypsin.

Copyright © 2002 John Wiley & Sons, Ltd. 

Table 2. Predicted number of peptides[1] for ideal global tryptic digestions

| Organism | Peptides [1] | Unique[2] | ORF Coverage[3] | Cys-peptides[1] | Unique[2] | ORF Coverage[3] |
|---|---|---|---|---|---|---|
| *D. radiodurans* | 60 068 | 51.4% | 99.4% | 4906 | 87.2% | 66% |
| *E. coli* | 84 162 | 48.6% | 99.1% | 11 487 | 83.6% | 80% |
| *S. cerevisiae* | 194 39 | 33.9% | 98% | 27 483 | 72.7% | 84% |
| *C. elegans* | 527 863 | 20.9% | 96.6% | 108 848 | 52.5% | 92% |

[1]Peptides or Cys-peptides in mass range of 500 to 4000 Da, assuming ideal trypsin cleavage specificity.
[2]Percent unique to $\pm 0.5$ ppm (by mass not using elution time).
[3]Percent of ORFs (or predicted proteins) covered by unique peptides.

will generally be obtained in each mass spectrum, requiring equivalent or less time than one MS/MS measurement, the increase in throughput is at the least equal to the average number of peptides in each spectrum. In practice the increase in either throughput or proteome coverage is even greater since the lower abundance peptides are often not analyzed by conventional MS/MS approaches, or require the need for additional time for extended ion accumulation or spectrum averaging to yield spectra of sufficient quality. Thus, the AMT approach provides increased sensitivity, coverage and throughput, and facilitates quantitative studies involving many analyses of different perturbations or time points.

The generation of most AMTs by this approach presently uses a two-stage process (Figure 2). The proteome sample is digested (e.g. with trypsin) and analyzed by high efficiency capillary LC-MS/MS using either a conventional (LCQ ion trap or Q-TOF) mass spectrometer operating in a data-dependent mode or using FTICR. The ion trap MS/MS measurements yield 'potential mass tags' (PMTs) that are subsequently validated as AMTs if the predicted peptide's accurate mass is observed using FTICR in a corresponding sample and at an equivalent elution time [13].

Ion trap MS/MS generated PMTs were initially identified using 'scores' produced by the SEQUEST search program based upon the similarity of the spectrum with a set of peaks predicted on the basis of the known most common peptide fragmentation processes. Due to the nature of the analysis, the results will invariably span the range from low scores where identifications are highly doubtful, to high scores where identifications are quite reliable, with no clear line of demarcation. If one uses only the highest scores for identification, fewer proteins will be identified, however, uncritical use of lower scores will result in many false identifications. Conventionally, many MS/MS spectra need to be manually examined so as to establish acceptable confidence for identifications. This process generally results in discarding a substantial fraction of the peptides identified with lower scores, and serves to increase the confidence to an extent that is difficult to quantify. In our approach the use of highly accurate mass measurements provides an additional, high quality 'test' for tentative peptide identifications that can be applied in the data analysis using software developed at our laboratory. A consequence of the automated validation of AMTs from PMTs is the increased confidence in the peptide identifications that results. Once a protein has been identified using AMTs, its subsequent identification (and quantitation) in other studies is based on FTICR measurements (and its elution time), which provide much greater sensitivity than the conventional MS instrumentation.

Once an AMT has been established, it can be used to confidently identify a specific protein in subsequent proteome studies. Without the need to re-establish the identity of a peptide using MS/MS analyses, multiple high throughput studies focused on measuring changes in relative protein abundances between two (or more) different proteomes are facilitated. In such comparative studies, stable isotope labeling methods can be used to provide a means to measure protein relative abundances, a process that also benefits from the resolution and sensitivity of the FTICR measurements.

Several strategies have been applied in our initial work with *D. radiodurans* to increase the number of AMTs so as to subsequently routinely allow lower-level proteins to be analyzed by this approach [13]. First, samples were analyzed several times using the same capillary LC-MS/MS strategy, but with different *m/z* ranges and with the 'exclusion' of parent ions that were previously selected for MS/MS, resulting in the selection of different peptides and

Copyright © 2002 John Wiley & Sons, Ltd. 

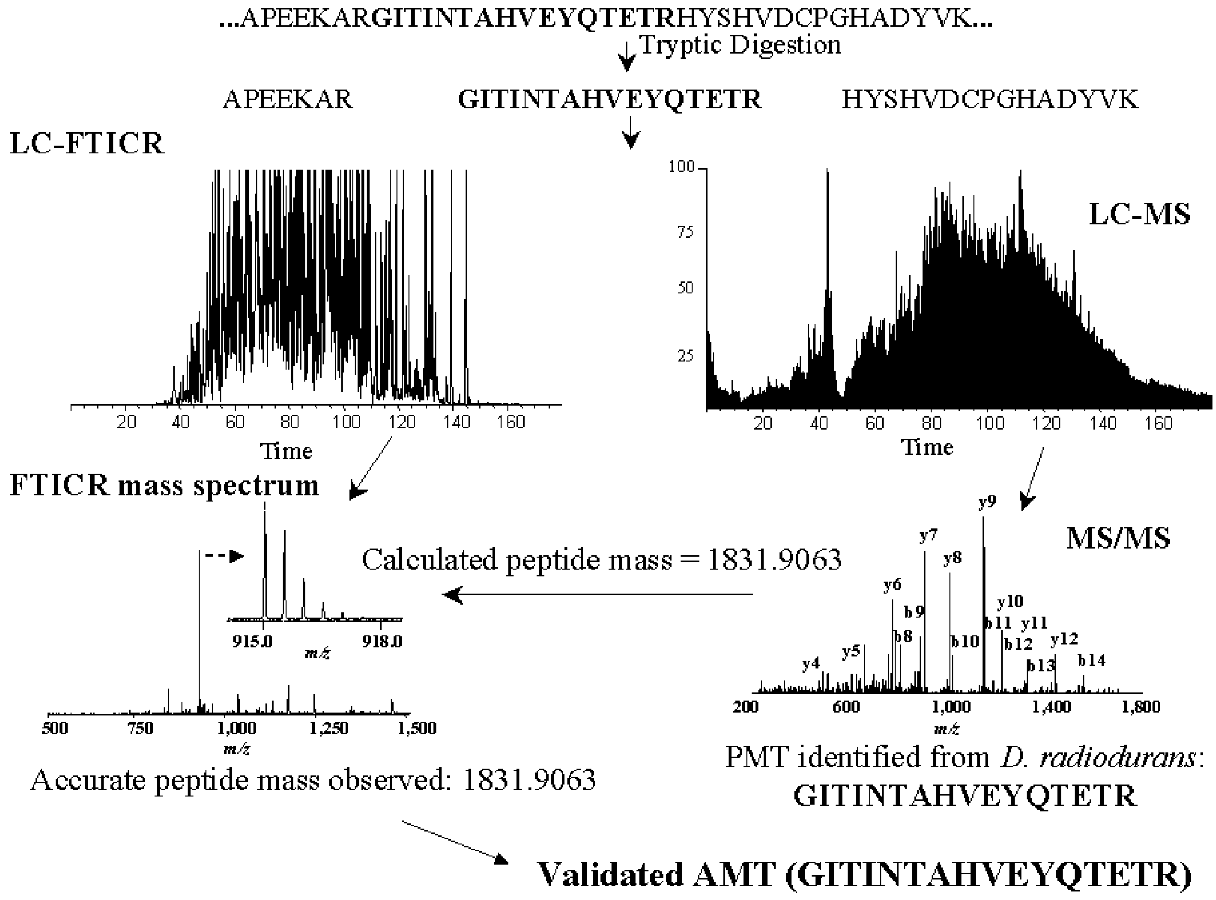

**Figure 2.** Experimental steps involved in establishing an accurate mass tag (AMT) illustrated by the identification of an AMT for elongation factor Tu (EF-Tu). Peptides are automatically selected for collisional induced dissociation (CID) and tentatively identified as a potential mass tag (PMT) using an automated search program (SEQUEST). In this example a tryptic peptide from EF-Tu (in bold) was identified by tandem MS (MS/MS) using an ion trap mass spectrometer. The accurate mass of this PMT was calculated based on its sequence and its elution time recorded. In the second stage, the same proteome sample is analyzed under the same LC-MS conditions using a high-field FTICR mass spectrometer. An AMT is established when a peptide eluting at the same time and corresponding to the calculated mass (e.g. within 1 ppm) of the PMT identified in the first stage is observed. This peptide is then considered an AMT for EF-Tu for *D. radiodurans* and functions as a biomarker to identify this particular protein in all subsequent experiments generation of additional PMTs. Beyond variations in instrumental approaches, proteome samples extracted from cells harvested at different growth phases (i.e. mid-log, stationary phase, etc.) or cultured under a variety of different conditions (i.e. nutrients, perturbations) were also analyzed. By varying growth conditions and harvesting stages, the potential pool of PMTs increases significantly since the absolute number of proteins collectively present in the different samples is significantly greater than the number expressed by the organism under a single growth condition. Finally, since any additional sample fractionation will increase the overall dynamic range achievable, peptide fractions were also analyzed after first being separated off-line by ion exchange chromatography, and which again results in the generation of large numbers of additional PMTs for peptides that would otherwise have too low abundance for conventional MS/MS analyses. The PMT generation efforts continued for *D. radiodurans* for over 200 different ion trap MS/MS runs, and until the rate of generation of novel PMTs decreased significantly. Since the analysis procedure can be totally automated, this

Copyright © 2002 John Wiley & Sons, Ltd. 

corresponds to a one-time effort requiring approximately three weeks using a single ion trap instrument, and additional experience should significantly reduce the number of runs required for PMT generation. It should be noted that any number of alternative sample fractionation and analysis strategies can be preformed to increase the number of PMTs and AMTs generated, and that the extra efforts at this stage are rewarded by the ability to subsequently make more comprehensive proteome measurements.

Analyses using *D. radiodurans* cultured under a number of different growth conditions, typically resulted in the detection of 20 000 to >50 000 peptides by capillary LC-FTICR analysis in each analysis (Figure 3). Using capillary LC with MS/MS measurements (including ion trap MS/MS measurements that generated >9000 PMTs), a total of 6997 peptides were validated as *bona fide* AMTs. These AMTs provide confident identification of 1910 predicted proteins (with an average of >3 AMTs per protein), covering ∼61% of the predicted proteome and spanning every category of predicted protein function from the annotated genome.

Proteome measurements often involve comparing protein abundances between two cellular populations resulting from, for example, some insult or perturbation. The predominant method for measuring changes in protein expression levels using

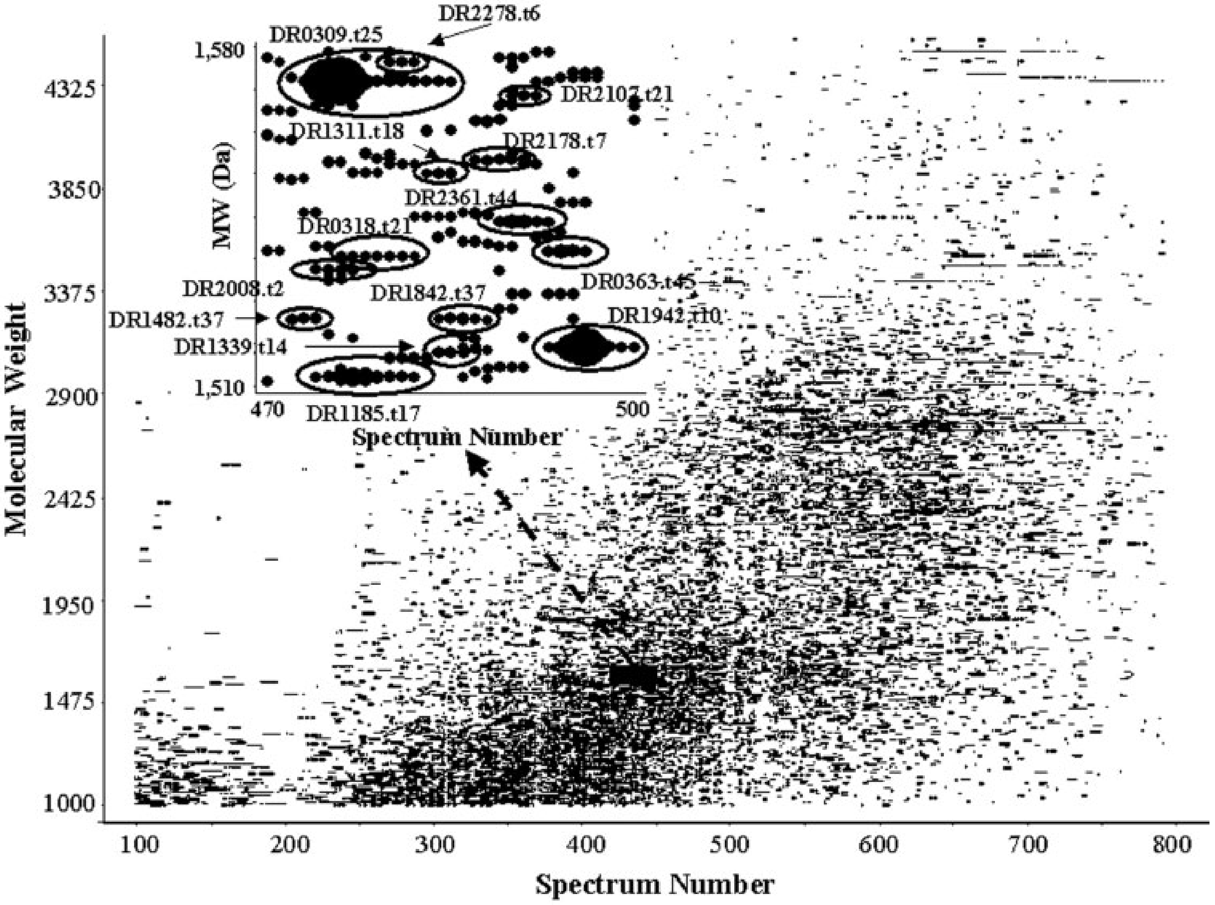

**Figure 3.** Two-dimensional display of peptides based on their molecular weight (MW) and elution order (i.e. FTICR spectrum number) and identified AMTs from Deinococcus radiodurans. The circled spots in the inset show spots that were identified as AMTs in the enlarged region. The spots are labeled based on their annotation within the organism's genome sequence (i.e. DR0309; elongation factor Tu) and the tryptic peptide of the protein that was identified (i.e. t25; the $25^{th}$ tryptic peptide counting from the amino terminus, based on complete digestion)

Copyright © 2002 John Wiley & Sons, Ltd.

current proteomic technology is to compare the intensities of the corresponding 2D PAGE spots. Attempts to infer absolute peptide abundances based upon MS signal intensities can be problematic for reasons that include variations in ionization efficiencies and losses during sample preparation and separations, and while useful for large differences in abundances, are unsuited to study more subtle variations. The generation and use of AMTs enables high throughput and high precision expression studies based upon stable-isotope labeling by directly comparing two proteomes in the same analysis (e.g. utilizing a 'reference proteome' to which perturbed systems are compared). A stable-isotope labeled reference proteome, for example, provides an effective internal standard for each protein, and hence their tryptic peptides, allowing changes in protein abundances based upon the relative abundances of AMTs to be assessed, potentially to precisions better than 10% [5,7,8]. While such measurements require both versions of the protein or peptide to be present, it should be feasible to combine this information with absolute peak intensity data to provide less precise abundances for cases where only one peptide is detected, and to also establish approximate absolute abundances (albeit, with less precision).

The large variation of protein relative abundances having potential biological significance in mammalian systems ($>6$ orders of magnitude) presents a major challenge for proteomics. We have recently developed a new Dynamic Range Enhancement Applied to Mass Spectrometry (DREAMS) approach that provides the basis for a significant gain in the coverage of proteomic measurements [1]. The DREAMS methodology involves acquisition of sets of mass spectra during the non-selective accumulation, in which each spectrum is followed by software-controlled selection of the most abundant ion peaks based on their quadrupole secular frequencies and then selective rf-only ejection of the most abundant species prior to external accumulation (for the next spectrum immediately following the non-selective 'normal' spectrum). This initial demonstration of the DREAMS FTICR method generated two data sets comprising spectra for the detected peptide isotopic distributions from the non-selective and selective DREAMS accumulations. It was found that the number of peptides detected with the alternating sequences (30 771 after subtraction of species detected in both) was greater by about 35% than that acquired using the non-selective ion accumulation (22 664). The same methodology was subsequently applied with data-dependent selective ion ejection of the two and three most abundant ion species. A 40% increase in the number of peptides was achieved when combining the non-selective ion accumulation with data-dependent selective ion ejection of the three most abundant ion species [1]. We believe that the DREAMS FTICR technology is an important component of an approach that provides the basis for a significant gain in the coverage of proteomic measurements.

While much remains to be done to refine and fully establish the high throughput potential of this approach, it clearly offers significant advantages in throughput, dynamic range and the completeness of proteome coverage. The approach described generates enormous quantities of data, which the initial work has only partly exploited. However, one can anticipate that this situation will change rapidly as additional and more powerful data analysis tools are implemented.

### Acknowledgement

The contributions of Ljiljana Pasa Tolic, Mary Lipton, Gordon Anderson, Kim Hixson, Ron Moore, Rui Zhao, Mike Belov, Christophe Masselon, David Anderson, Nikola Tolic, Nicolas Angell, and Yufeng Shen to the work reviewed here are gratefully acknowledged. We thank the U.S. Department of Energy Office of Biological and Environmental Research for long time support of the *D. radiodurans* research and the FTICR technology development, as well as the National Institutes of Health, through NCI (CA81654), NINDS (NS39617) and NCRR (RR12365) for support of portions of this work. Pacific Northwest National Laboratory is operated by Battelle Memorial Institute for the U.S. Department of Energy under contract DE-AC06-76RLO 1830.

### References

1. Belov ME, Anderson GA, Angell NH, *et al*. 2001. Dynamic range expansion applied to mass spectrometry based on data-dependent selective ion ejection in capillary liquid chromatography Fourier transform ion cyclotron resonance for enhanced proteome characterization. *Anal Chem* **73**: 5052–5060.
2. Conrads TP, Alving K, Veenstra TD, *et al*. 2001. Quantitative analysis of bacterial and mammalian proteomes using a combination of cysteine affinity tags and 15N-metabolic labeling. *Anal Chem* **73**: 2132–2139.
3. Conrads TP, Anderson GA, Veenstra TD, Pasa-Tolic L,

Copyright © 2002 John Wiley & Sons, Ltd. 

Smith RD. 2000. Utility of accurate mass tags for proteome-wide protein identification. *Anal Chem* **72**: 3349–3354.

4. Gygi SP, Corthals GL, Zhang Y, Rochon Y, Aebersold R. 2000. Evaluation of two-dimensional gel electrophoresis-based proteome analysis technology. *Proc Natl Acad Sci U S A* **97**: 9390–9395.
5. Gygi SP, Rist B, Gerber SA, *et al.* 1999. Quantitive analysis of complex protein mixtures using isotope-coded affinity tags. *Nat Biotechnol* **17**: 994–999.
6. , Li L, Masselon C, Anderson GA, *et al.* 2001. High-throughput peptide identification from protein digests using data-dependent multiplexed tandem FTICR mass spectrometry coupled with capillary liquid chromatography. *Analy Chem* **73**: 3312–3322.
7. Oda Y, Huang K, Cross FR, Cowburn D, Chait BT. 1999. Accurate quantitation of protein expression and site-specific phosphorylation. *PNAS* **96**: 6591–6596.
8. Pasa-Tolic L, Jensen PK, Anderson GA, *et al.* 1999. High throughput proteome-wide precision measurements of protein expression using mass spectrometry. *J Amer Chem Soc* **121**: 7949–7950.
9. Shen Y, Tolic N, Zhao R, *et al.* 2001. High-throughput proteomics using high efficiency multiple-capillary liquid chromatography with on-line high performance ESI FTICR mass spectrometry. *Anal Chem* **73**: 3011–3021.
10. Shevchenko A, Jensen ON, Podtelejnikov AV, *et al.* 1996. Linking genome and proteome by mass spectrometry: large-scale identification of yeast proteins from two dimensional gels. *Proc Natl Acad Sci U S A* **93**: 14440–14445.
11. Shevchenko A, Wilm M, Vorm O, Mann M. 1996. Mass spectrometric sequencing of proteins from silver stained polyacrylamide gels. *Anal Chem* **68**: 850–858.
12. Smith RD. 2000. Probing proteomes– Seeing the whole picture? *Nature Biotech* **18**: 1041–1042.
13. Smith RD, Anderson GA, Lipton MS, *et al.* 2002. An accurate mass tag strategy for quantitative and high throughput proteome measurements. *Proteomics* (in press).
14. Wilm M, Shevchenko A, Houthaeve T, *et al.* 1996. Femtomole sequencing of proteins from polyacrylamide gels by nano-electrospray mass spectrometry. *Nature* **379**: 466–469.
15. Yates JRI, Speicher S, Griffin PR, Hunkapiller T. 1993. Peptide mass maps: a highly informative approach to protein identification. *Anal Biochem* **214**: 397–408.

Copyright © 2002 John Wiley & Sons, Ltd. 
